# Supplementary material for: A Tale of 12 Tails: Katanin Severing Activity Affected by Carboxy-Terminal Tail Sequences
Source: Biomolecules. 2023 Mar 30;13(4):620. doi: 10.3390/biom13040620 (PMC10136189; doi:10.3390/biom13040620)
Supplement: Supplementary file 1 [file biomolecules-13-00620-s001.zip › SI/Lindsay-Biomolecules-SI-R2.pdf]

A Tale of 12 Tails: Katanin Severing Activity Affected by Carboxy-Terminal Tail Sequences

K. Alice Lindsay [1], Nedine Abdelhamid[1], Shehani Kahawatte[2], Ruxandra I. Dima[2], Dan L. Sackett[3], Tara M. Finegan[1], and Jennifer L. Ross [1,*]

Supplemental Information

This supplemental information contains 12 tables with fits to the data given in the main document, one supplemental figure, and supplemental movies representative of each data set and a table with information for each movie.

Table S1. Loss of microtubule polymer fits for data in Figure 1.

**Figure**	**Fit equation**	M(∞)	**A**	τ (s)	R^2	X^2
Figure 1Aii	M(t) = M(∞) + A*exp*(- t/τ)	0.020 ± 0.004	1.77 ± 0.04	46 ± 1	0.993	0.0292
Figure 1Bii	M(t) = A(1-t/τ)	NA	0.994 ± 0.004	528 ± 4	0.991	0.0123
Figure 1Cii	M(t) = A(1-t/τ)	NA	1.013 ± 0.001	1220 ± 10	0.990	0.00426

Table S2. Loss of microtubule polymer fits for data in Figure 2.

**CTT**	**Concentration (μM)**	**Fit equation**	M(∞)	**A**	τ (s)	R^2	X^2
Free tubulin, Figure 2Ai	0.0	M(t) = M(∞) + A*exp*(- t/τ)	0.009 ± 0.004	1.84 ± 0.04	43.7 ± 0.9	0.993	0.0317
	0.5	M(t) = M(∞) + A*exp*(- t/τ)	0.040 ± 0.005	2.9 ± 0.2	31 ± 1	0.979	0.045
	0.75	M(t) = M(∞) + A*exp*(- t/τ)	0.221 ± 0.002	2.11 ± 0.04	59.7 ±0.9	0.998	0.0026
	1.0	M(t) = M(∞) + A*exp*(- t/τ)	0.133 ± 0.002	1.23 ± 0.02	45.7 ±0.7	0.997	0.0100
	1.5	M(t) = A(1-t/τ)	NA	0.813 ± 0.002	517 ±3	0.996	0.0039
	2.0	M(t) = A(1-t/τ)	NA	1.013 ± 0.001	1220 ±10	0.990	0.0043

alpha1 (TUBA1A) Figure 2Aii	0.0	$M(t) = M(\infty) + A\textit{exp}(- t/\tau)$	0	6.8 ±0.3	32.6 ±0.7	0.994	0.0088
	0.5	$M(t) = M(\infty) + A\textit{exp}(- t/\tau)$	0	2.18 ±0.03	65.367 ±0.5511	0.997	0.010
	0.75	$M(t) = M(\infty) + A\textit{exp}(- t/\tau)$	0	1.330 ±0.01	220.62 ± 3	0.991	0.016
	1.0	$M(t) = M(\infty) + A\textit{exp}(- t/\tau)$	0.511 ±0.005	1.731 ±0.07	31.847 ± 3	0.996	0.0037
	1.5	$M(t) = A(1-t/\tau)$	NA	1.316 ±0.007	354.71 ± 2	0.995	0.0097
	2.0	$M(t) = A(1-t/\tau)$	NA	1.0293 ±0.002	1190.8 ± 20	0.984	0.0046
beta (TUBB) Figure 2Aiii	0.0	$M(t) = M(\infty) + A\textit{exp}(- t/\tau)$	0	3.38 ±0.08	47.786 ±0.7	0.994	0.037
	0.5	$M(t) = A(1-t/\tau)$	NA	1.182 ±0.002	410.6 ±0.9	0.999	0.0028
	0.75	$M(t) = M(\infty) + A\textit{exp}(- t/\tau)$	0	2.60 ±0.06	66 ±1	0.992	0.0367
	1.0	$M(t) = A(1-t/\tau)$	NA	1.133 ±0.004	1410 ±30	0.960	0.0062
	1.5	$M(t) = A(1-t/\tau)$	NA	1.076 ±0.003	1250 ± 20	0.983	0.0027
	2.0	$M(t) = A(1-t/\tau)$	NA	1.076 ±0.002	1370 ±20	0.979	0.0040

Supplemental Table S3. Severing Rate vs. Maximum GFP Intensity for data in Figure 2F. Fits to a linear fit equation: y = mx +b.

**CTT**	**m**	b	$\mathbf{R^2}$	$\boldsymbol{X^2}$
Free tubulin (gray line)	0.021 ± 0.005	0.002 ± 0.003	0.859	0.0000649
Alpha1 (red line)	0.05 ± 0.01	-0.022 ± 0.003	0.817	0.0000511
Beta (blue line)	0.04 ± 0.01	-0.019 ± 0.008	0.876	0.0000388

Supplemental Table S4. Loss of microtubule polymer fits for data in Figure 3.

**CTT**	**Concentration (μM)**	**Fit equation**	M(∞)	**A**	*τ* (s)	R^2	X^2
Detyrosinated Alpha (Alpha1-Y)	0.0	M(t) = M(∞) + A*exp*(- t/τ)	0	1.89 ±0.05	19.5 ±0.4	0.995	0.0081
	0.5	M(t) = M(∞) + A*exp*(- t/τ)	0	4.4 ±0.5	18 ±1	0.969	0.0510
	0.75	M(t) = M(∞) + A*exp*(- t/τ)	0	13. ±2	10.0 ±0.5	0.986	0.0084
	1.0	M(t) = M(∞) + A*exp*(- t/τ)	0	2.85 ±0.07	30.7 ±0.4	0.996	0.0079
	1.5	M(t) = M(∞) + A*exp*(- t/τ)	0	1.56 ±0.02	85.7 ±0.9	0.995	0.0185
	2.0	M(t) = M(∞) + A*exp*(- t/τ)	0	1.020 ±0.002	545 ±4	0.995	0.0068
**Δ**2 Alpha1 (Alpha1-YE)	0.0	M(t) = M(∞) + A*exp*(- t/τ)	-0.020 ±0.007	1.16 ±0.01	59 ± 2	0.989	0.0935
	0.5	M(t) = M(∞) + A*exp*(- t/τ)	0	4.3 ±0.2	23.3 ± 0.5	0.989	0.0277
	0.75	M(t) = M(∞) + A*exp*(- t/τ)	0	3.09 ±0.06	57.7 ±0.6	0.997	0.0074
	1.0	M(t) = M(∞) + A*exp*(- t/τ)	0	1.06 ±0.02	104 ±2	0.973	0.0690
	1.5	M(t) = A(1-t/τ)	NA	1.039 ±0.005	328 ± 2	0.995	0.0134
	2.0	M(t) = A(1-t/τ)	NA	1.031 ±0.002	578 ± 2	0.998	0.0018

Supplemental Table S5. Severing Rate vs. Maximum GFP Intensity for data in Figure 3F. Fits to a linear fit equation: y = mx +b.

CTT	m	b	R^2	X^2
Alpha1 (red line)	0.05 ± 0.01	-0.022 ± 0.003	0.817	0.0000511
Detyrosinated Alpha1 (light orange line)	0.10 ± 0.03	-0.05 ± 0.04	0.560	0.0027
**Δ**2 Alpha1 (dark orange line)	0.06 ± 0.03	-0.03 ± 0.03	0.500	0.000568

Supplemental Table S6. Loss of microtubule polymer fits for data in Figure 4.

CTT	Concentration (μM)	Fit equation	M(∞)	A	τ (s)	R^2	X^2
beta2a (TUBB2A)	0.0	$M(t) = M(\infty) + A\,exp(-t/\tau)$	0.020 ±0.02	1.68 ±0.04	32.1 ±0.7	0.991	0.0201
	0.5	$M(t) = M(\infty) + A\,exp(-t/\tau)$	0.021 ±0.002	3.7 ±0.1	19.3 ±0.4	0.993	0.0140
	0.75	$M(t) = M(\infty) + A\,exp(-t/\tau)$	0.003 ±0.005	1.22 ±0.02	44 ±1	0.985	0.0709
	1.0	$M(t) = M(\infty) + A\,exp(-t/\tau)$	0	1.53 ±0.04	16.0 ±0.4	0.991	0.0171
	1.5	$M(t) = A(1-t/\tau)$	NA	1.025 ±0.002	1310 ±10	0.988	0.0018
	2.0	$M(t) = A(1-t/\tau)$	NA	0.998 ±0.002	1770 ±40	0.953	0.0010
beta3 (TUBB3)	0.0	$M(t) = M(\infty) + A\,exp(-t/\tau)$	0	3.4 ±0.3	8.9 ±0.4	0.988	0.0117
	0.5	$M(t) = M(\infty) + A\,exp(-t/\tau)$	0.037 ±0.04	1.76 ±0.07	20.2 ±0.7	0.974	0.0834
	0.75	$M(t) = M(\infty) + A\,exp(-t/\tau)$	0.008 ±0.002	4.0 ±0.1	18.6 ±0.4	0.993	0.0192
	1.0	$M(t) = M(\infty) + A\,exp(-t/\tau)$	0.063 ±0.001	1.40 ±0.01	42.1 ±0.4	0.999	0.0037

	1.5	$M(t) = M(\infty) + A\mathit{exp}(- t/\tau)$	0.017 ±0.003	4.2 ±0.2	41.6 ±0.8	0.995	0.0128
	2.0	$M(t) = M(\infty) + A\mathit{exp}(- t/\tau)$	0.046 ±0.002	3.5 ±0.1	16.1 ±0.3	0.992	0.0133
beta4b (TUBB4b)	0.0	$M(t) = M(\infty) + A\mathit{exp}(- t/\tau)$	0	2.50 ±0.06	17.0 ±0.3	0.993	0.0141
	0.5	$M(t) = M(\infty) + A\mathit{exp}(- t/\tau)$	0.02 ±0.01	1.01 ±0.07	32 ±3	0.926	0.1305
	0.75	$M(t) = M(\infty) + A\mathit{exp}(- t/\tau)$	0	1.34 ±0.01	203 ±3	0.988	0.0548
	1.0	$M(t) = M(\infty) + A\mathit{exp}(- t/\tau)$	-0.045 ±0.005	1.63 ±0.02	83 ±1	0.998	0.0107
	1.5	$M(t) = A(1-t/\tau)$	NA	1.139 ±0.006	670 ±10	0.976	0.0147
	2.0	$M(t) = A(1-t/\tau)$	NA	1.149 ±0.004	559 ±4	0.994	0.0054

Supplemental Table S7. Severing Rate vs. Maximum GFP Intensity for data in Figure 4D. Fits to a linear fit equation: $y = mx + b$.

**CTT**	**m**	b	**R^2**	**X^2**
Beta (blue line)	0.04 ± 0.01	-0.019 ± 0.008	0.876	0.0000388
Beta2A (light blue line)	0.05 ± 0.02	0.00 ± 0.02	0.568	0.00142
Beta3 (dark blue line)	0.19 ± 0.08	-0.11 ± 0.07	0.568	0.00232
Beta4b (cyan line)	0.13 ± 0.04	-0.07 ± 0.03	0.806	0.000368

Supplemental Table S8. Loss of microtubule polymer fits for data in Figure 5.

CTT	Concentration (μM)	Fit equation	M(∞)	A	τ (s)	R^2	X^2
Alpha1-Y+F	0.0	$M(t) = M(\infty) + A\,exp(-t/\tau)$	0.009 ±0.003	1.49 ±0.03	43.5 ±0.9	0.993	0.0297
	0.5	$M(t) = M(\infty) + A\,exp(-t/\tau)$	0	4.4 ±0.5	18 ±1	0.969	0.0510
	0.75	$M(t) = M(\infty) + A\,exp(-t/\tau)$	0.015 ±0.003	2.43 ±0.09	27.1 ±0.7	0.991	0.0214
	1.0	$M(t) = M(\infty) + A\,exp(-t/\tau)$	0.026 ±0.003	1.77 ±0.03	46.4 ±0.9	0.996	0.0113
	1.5	$M(t) = M(\infty) + A\,exp(-t/\tau)$	0	2.55 ±0.07	43.6 ±0.8	0.992	0.0270
	2.0	$M(t) = M(\infty) + A\,exp(-t/\tau)$	0	1.42 ±0.01	140 ±1	0.997	0.0033
B1-A+Y	0.0	$M(t) = M(\infty) + A\,exp(-t/\tau)$	0	2.40 ±0.05	43.6 ±0.6	0.995	0.0190
	0.5	$M(t) = M(\infty) + A\,exp(-t/\tau)$	0.033 ±0.004	3.0 ±0.1	31.8 ±0.9	0.987	0.0455
	0.75	$M(t) = M(\infty) + A\,exp(-t/\tau)$	0	4.4 ±0.3	21.8 ±0.7	0.989	0.0163
	1.0	$M(t) = M(\infty) + A\,exp(-t/\tau)$	0	2.91 ±0.09	51.8 ±0.9	0.990	0.0403
	1.5	$M(t) = M(\infty) + A\,exp(-t/\tau)$	0	1.83 ±0.04	55 ±1	0.988	0.0438
	2.0	$M(t) = M(\infty) + A\,exp(-t/\tau)$	0.062 ±0.003	1.51 ±0.01	76 ±1	0.999	0.0049

Supplemental Table S9. Severing Rate vs. Maximum GFP Intensity for data in Figure 5E. Fits to a linear fit equation: y = mx +b.

CTT	m	b	R^2	X^2
Alpha1-Y+F (magenta line)	0.03 ± 0.04	0.00 ± 0.04	0.129	0.001133
Beta-A+Y (purple line)	0.03 ± 0.02	0.00 ± 0.02	0.262	0.000520

Supplemental Table S10. Loss of microtubule polymer fits for data in Figure 6.

CTT	Concentration (μM)	Fit equation	M(∞)	A	τ (s)	R^2	X^2
E10	0.0	M(t) = M(∞) + A*exp*(- t/τ)	0	1.45 ±0.03	31.4 ±0.6	0.993	0.0196
	0.5	M(t) = M(∞) + A*exp*(- t/τ)	0	1.71 ±0.03	36.3 ±0.6	0.993	0.0374
	0.75	M(t) = M(∞) + A*exp*(- t/τ)	0	1.40 ±0.01	60.7 ±0.6	0.998	0.0049
	1.0	M(t) = M(∞) + A*exp*(- t/τ)	0	1.90 ±0.05	52.0 ±0.9	0.990	0.0337
	1.5	M(t) = A(1-t/τ)	NA	1.032 ±0.003	851 ±9	0.985	0.0142
	2.0	M(t) = A(1-t/τ)	NA	1.010 ±0.004	447 ± 3	0.992	0.0279
D10	0.0	M(t) = M(∞) + A*exp*(- t/τ)	0	1.86 ±0.04	40.9 ± 0.6	0.994	0.0175
	0.5	M(t) = M(∞) + A*exp*(- t/τ)	0.024 ±0.003	1.51 ±0.01	55.4 ± 0.7	0.998	0.0106
	0.75	M(t) = M(∞) + A*exp*(- t/τ)	0	1.86 ±0.03	53 ± 0.6	0.996	0.0185
	1.0	M(t) = M(∞) + A*exp*(- t/τ)	0	1.115 ±0.006	173 ±1	0.997	0.0096
	1.5	M(t) = A(1-t/τ)	NA	1.053 ±0.003	586 ± 3	0.996	0.0051
	2.0	M(t) = A(1-t/τ)	NA	1.080 ±0.004	566 ± 4	0.990	0.0138

Supplemental Table S11. Severing Rate vs. Maximum GFP Intensity for data in Figure 6E. Fits to a linear fit equation: y = mx +b.

**CTT**	**m**	b	**R^2**	**X^2**
E10 (light green line)	0.02 ± 0.02	0.00 ± 0.01	0.104	0.0004902
D10 (dark green line)	0.04 ± 0.01	-0.02 ± 0.01	0.678	0.0001558

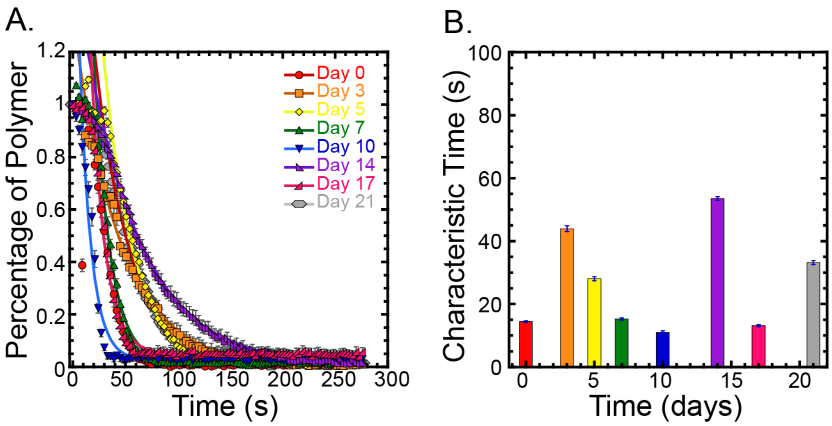

Supplemental Figure S1. Katanin Activity fluctuates over time. (A) One preparation of katanin was stored in -80°C in 50% glycerol in small aliquots. Day 0 denotes the same day as the preparation. The katanin was used for loss of polymer assays with 680 nM katanin, 2 mM ATP, and oxygen scavenging system, as described in the methods. The data is fit with an exponential decay (see Supplemental Table S12). (B) The best fits for the characteristic decay of the data plotted as a bar plot over the course of 21 days. As shown, there are day-to-day fluctuations in the activity of the katanin. We have also observed what appeared to be loss of polymer with other preparations. Some preparations (about 1/3) do not sever well and need a high concentration of katanin to work.

Supplemental Table S12. Loss of microtubule polymer fits for data in Supplemental Figure S1.

**Data set**	**Fit equation**	M(∞)	**A**	τ (s)	**R^2**	**X^2**
Day 0	M(t) = M(∞) + A*exp*(- t/τ)	0.007 ± 0.001	3.8 ± 0.2	14.4 ± 0.3	0.993	0.0107
Day 3	M(t) = M(∞) + A*exp*(- t/τ)	0	1.34 ± 0.02	44.0 ± 0.9	0.993	0.0344
Day 5	M(t) = M(∞) + A*exp*(- t/τ)	0.007 ± 0.003	3.3 ± 0.1	28.0 ± 0.6	0.992	0.0230
Day 7	M(t) = M(∞) + A*exp*(- t/τ)	0.013 ± 0.002	4.3 ± 0.2	15.3 ± 0.3	0.990	0.0221
Day 10	M(t) = M(∞) + A*exp*(- t/τ)	0.026 ± 0.004	2.1 ± 0.1	11.0 ± 0.5	0.955	0.0880
Day 14	M(t) = M(∞) + A*exp*(- t/τ)	0.003 ± 0.002	1.57 ± 0.01	53.5 ± 0.6	0.998	0.0073
Day 17	M(t) = M(∞) + A*exp*(- t/τ)	0.048 ± 0.002	4.2 ± 0.2	13.1 ± 0.4	0.986	0.0259
Day 21	M(t) = M(∞) + A*exp*(- t/τ)	0.011 ± 0.003	2.22 ± 0.05	33.2 ± 0.6	0.994	0.0202

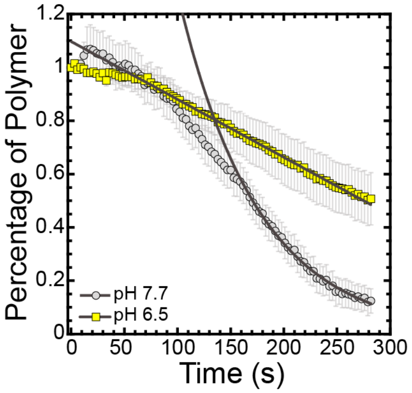

Supplemental Figure S2. pH affects katanin activity. Experiments are performed at pH 7.7 in the main paper. When the pH is shifted to 6.5, the activity decreases significantly. Fit equations are given in Supplemental Table S13.

Supplemental Table S13. Loss of microtubule polymer fits for data in Supplemental Figure S2.

**Data set**	**Fit equation**	M(∞)	**A**	τ (s)	R^2	X^2
pH 7.7	$M(t) = M(\infty) + A\,exp(-t/\tau)$	0	4.8 ± 0.1	75.4 ± 0.8	0.996	0.00284
pH 6.5	$M(t) = A(1-t/\tau)$	NA	1.097 ± 0.003	505 ± 2	0.997	0.00336
